# The Self of Adolescents with Autism Spectrum Disorder or Attention Deficit Hyperactivity Disorder: A Qualitative Study

**DOI:** 10.1007/s10803-020-04653-7

**Published:** 2020-08-24

**Authors:** Fumi Hanai, Miho Narama, Koji Tamakoshi

**Affiliations:** 1 Department of Nursing, Nagoya University Graduate School of Medicine, Japan, 1-20-1, Daikominami, Higashiku, Nagoya, Aichi Japan; 2 Department of Nursing, Kyoto Tachibana University, 34, Yamada-chou, Yamasinaku, Kyoto, Japan

**Keywords:** Self-development, Adolescent, Autism spectrum disorder, Attention deficit hyperactivity disorder

## Abstract

Self-development is a central developmental issue in adolescence, there are few studies describing the experiences related to the self in adolescents with autism spectrum disorder (ASD) or attention deficit hyperactivity disorder (ADHD). We conducted semi-structural interviews with 14 adolescents with ASD and three with ADHD to describe the self. As a result of inductive continuous comparison analysis, three concepts “Interest in self and self-realization”, “Intentionality and self-transformation”, “Unrealized/unnoticed self” were generated. It was suggested that the characteristic perceptions may tend to have difficulty realizing subjective selves.Otherwise, most adolescents realized various sense of self through interaction with others. Nurses should know adolescents’ inner world and share their emotions related to their self-recognition in order to provide care that meets important youth developmental needs.

## Background: Adults and Adolescents with ASD or ADHD

Autism spectrum disorder (ASD) is a neurodevelopmental disability defined by diagnostic criteria that include deficits in social communication and social interaction, and the presence of restricted, repetitive patterns of behavior, interests, or activities that can persist throughout life (APA 2013). Attention deficit/hyper-activity disorder (ADHD) is defined by the core symptoms of hyperactivity, impulsivity, and inattention (APA 2013). Although many ASD and ADHD people have high intellectual abilities, they are prone to difficulties in relationship and emotional problems, so they require social and emotional support for a long time (Morioka and Yamamoto 2014; Shibata et al. 2011; Soo et al. 2018).

The prevalence of children with ASD or ADHD is estimated to be about 5 to 8% in the United States and South Korea (Centers for Disease Control and Prevention 2019a, b; Zablotsky et al. 2015; Kim et al. 2011; Song et al. 2018). In Japan, the enrollment rate of children who are considered to require special consideration for learning and behavior is about 6.5% (Ministry of Education 2012). In adolescence, young people gradually shift their allegiance from home to society and develop an ego identity that is multifaceted but integrated and consistent (Hattori 2010). Adolescents often experience confusion, anxiety, depression, and increasing tension with their parents while establishing their identity and independence. It can be a time of instability, but currently there is not sufficient comprehensive, a long-term support for adolescents with developmental disorders. There are very few specialized outpatients and wards for adolescents with developmental disabilities. There are only 31 medical facilities with child psychiatric wards (Japanese Council of Child and Adolescent Mental Institution 2020), and few hospital with day-care program for adolescents (Kinoshita et al. 2017).

There are various theories about the self. Mead (1987) defined the self as “a subjective ego” that perceives and feels, and as “an objective ego” that is targeted, and stated that self reflects the type of behavioral activity approved by social groups. Erikson defined the self as the process of selecting and structuring the self-image, which is formed through interaction with others, with the product being ego identity (Kounogi 1971). The self is formed through interaction with others, and includes the subject that perceives and recognizes, and the object captured by the subject.

Stern (1989) inferred that infants have the “sense of an emergent self” by the first 2 months, the “sense of a core of self” between 2 and 6 months, the “sense of a subjective self” between seven and 15 months, and the “sense of a verbal self” in 15–18 months. These “senses of self” appeared sequentially, and once formed, they are active for a lifetime. Stern also said that “senses of self” is the basis of subjective experience in human social development. Human relationships are the foundation of life and affect health and self-worth (Brown and Lohr 1987). In other words, it is necessary to pay attention to self when considering support for adolescents with developmental disabilities who are likely to have difficulty in human relationships.

There are few studies on self-development in children and adolescents with ASD or ADHD. They tend to perceive themselves negatively as “I am who cannot do well” (Miyamoto 2010) and to have low self-esteem (Nishimura 2009; Tominaga et al. 2017) There is a report about a unique self for children with ASD. They evaluate their own sociality as low (“I have few friends”), but that does not necessarily imply low self-esteem (Oka and Ono 2010). For children with ADHD, their self-evaluation of sociality and behavior is lower than that of children with typical development (Nakayama and Tanaka 2008), and higher symptom scores correlate to lower self-esteem scores (Edbom 2008). However, Takiyoshi and Tanaka (2009) have pointed out that development measures were created for children with typical development and may not sufficiently capture the characteristics of children with developmental disabilities. There is a limit to the ability of quantitative measures to describe the subjective and multifaceted self.

Sugiyama (2000, 2004) analyzed autobiography of adults with ASD and pointed out that they may have unique perceptual experiences and self-structure but lack of experience of integration with caregivers, and their self-other experiences may not overlap in interpersonal interaction. About ADHD, there are reports such as “It is difficult for both men and women to have a stable self” (Krueger et al. 2001), in adolescence, negative self-image become evident to others (Nishida 2014). Adolescents with ASD or ADHD may have their own characteristic self, but the specific experiences of the regarding self are not sufficiently clear.

Wallon (1965) said the ego’s consciousness evolves in emotional movements and relationships with others. In daily lives, it is not only cognition but emotion that is important when people interact with others (Beppu 2013). Stern focused not only on observed empirical behavior, but also on infants’ subjective experiences through interaction with parents; he defined parents’ imitation of infants’ behavior and the expression of shared emotions as “affect attunement.” To clarify the development of self, it is necessary to pay attention to both emotions and interactions.

In adolescence the experience of interaction within the peer group provides not only an emotional base but also a realistic evaluation of one’s ability, and integration of viewpoints of the self and others, and an opportunity to learn cooperation and to escape from self-centeredness (Kin and Hosokawa 2005). However, adolescents with ASD or ADHD are more likely to have difficulty in building friendships and sharing emotional involvement with others (Sonobe 2014). This study suggests that it may be useful to consider support for those who are more likely to have difficulties with the interactions required for self-development. The purpose of the study is to describe the self of adolescents with ASD or ADHD by focusing on their subjective experiences.

## Methods

### Participants

Participants were recruited through Japanese child psychiatrists whose patients had ASD or ADHD. For inclusion, study participants had to be: (a) adolescents who had a previous known diagnosis of ASD or ADHD, (b) 13 to 25 years old, (c) Japanese speakers, (d) without intellectual disabilities, (e) whose mental and physical states were stable.

### Procedure

The researcher asked the primary care physician to select. In order to make the instrument more visually comprehensible, we made two types with simple and concise expressions, taking into consideration the characteristics of the disorder and the developmental stage. The researcher contacted the people who wanted to participate to, ask about the interview date and place and desire to parents’ attend.

The first author is doctoral student and has seven years of experience in providing nursing care to adolescents with developmental disabilities and, conducted all interviews. Demographic data were collected with the original questionnaire before the interview. The survey was conducted between September 2016 and September 2017. Interviews were semi-structured, using open-ended questions. The framework was not prepared by the researcher, because the purpose of the study is to know the self from the participant’s points of view. A guide to questions can be found in Table 1.

**Table 1:** Guide to questions

**Q1. Please tell us about your first interest in yourself.**
(1) How old were you when you started to be interested in yourself?
(2) How did you become interested in yourself and what was your feeling and experience at that time?
(3) At that time, how did you view yourself?
(4) Please tell us about the events that left the impression at that time
(5) If there are events that have influenced your perception, please let us know
**Q2. Please tell us about current yourself and self-perception**
(1) How do you view yourself today?
(2) Please tell us about your feelings and experiences at the moment when you started to understand your current perspective
(3) Please tell us about the events that left the impression at that time
(4) If there are any events that have influenced your current perception, please let us know
**Q3. Please tell us about your changes in yourself and self-perception**
(1) Has your attitude changed since you started to be interested in yourself?
(2) How old were you when this change occurred?
(3) Please tell us how you changed your perception and your feelings and experiences at that time
(4) Please tell us about the events that left the impression at that time
(5) How has your attitude changed?
(6) If there is an event that has affected the change of your perception, please let us know
**Q4. If you missed telling us something during the interview, please let us know**

### Ethical Considerations

All procedures were approved by the University Bioethics Committee and by the Research Cooperation Facility. The author also made prior arrangements to refer participants in need to appropriate resources in case the need for professional support was identified during the interview. However, no such referrals were necessary.

### Theoretical Approach

Adolescents with developmental disorders may experience different sensations and perceptions of self than adolescents without developmental disorders, and we anticipated encountering phenomena that cannot be explained by existing theories. Therefore, this research was based on the phenomenology proposed by Husserl, with the intention of exploring the diversity of consciousness and the human world (Burns and Grove 2007). In order to describe participants’ experiences without prejudice as much as possible, we decided not to prepare a conceptual framework but instead to “bracket” the subjectivity, values, and experiences of researchers and work on analysis. Inductive methods and descriptive search designs were selected.

16 Interviews were audiotaped, and transcribed verbatim. Only one participant did not consent to recording, so we made a record from the notes of the content of the interview. The first author read the transcripts iteratively and extracted descriptions about self and interactions, summarizing them in meaningful and concise sentences, and coding them. We performed continuous comparison analysis, examined similarities and differences between codes, and generated subcategories. We examined similarities and differences between subcategories to generate more abstract categories and concepts. In the process of interpretation and analysis, we repeatedly reviewed whether there was any excess or deficiency, checking with a pediatric nursing researcher and to ensure validity. Another pediatric nursing researcher confirmed that the names and contents of concepts, categories, and subcategories were appropriate. After confirmation, we finally extracted three concepts.

## Results

Participants were aged 13–24 (mean age 17.4 years), 13 were male and 4 were female. Interview times lasted between 20 and 142 min (70 min on average) and 3 participants were accompanied by their mothers (see Table 2). Three concepts emerged: 【Interest in self and self-realization】, 【Intentionality and self-transformation】, and 【Unrealized or unnoticed self】. The concepts are explained below using categories and specific narratives. Boldface type indicates the concept, boldface italics indicates category. Narratives are indented, and parenthetical information follows each narrative listing case letter, age, and gender.

**Table 2:** Characteristics of participants

Case	Age	Sex	Diagnosis	Affiliation
A	19	M	ASD	College student
B	17	M	ASD	High school student
C	16	M	ADHD	High school student
D	24	F	ADHD, bronchial asthma	Part time job
E	24	M	ASD	Part time job
F	13	M	ASD	Junior high school student
G	13	F	ASD	Junior high school student
H	18	M	ASD	High school student
I	18	F	ASD	High school student
J	15	F	ASD	Junior high school student
K	17	M	ASD	High school student
L	18	M	ASD	High school student
M	16	M	ADHD	High school student
N	18	M	ASD	High school student
O	16	M	ASD, irritable colitis	High school student
P	17	M	ASD, food allergies, bronchial asthma	High school student
Q	18	M	ASD	High school student

**Interest inself and self-realization**

***Focusing on and thinking about myself***

Participants began to be consistently interested in themselves. The age for this varied from early childhood to adulthood.

I have not been interested in myself so far [laugh]. Re,,,cently, maybe, I just started to get interested in myself. (D/24, f)

Some participants came to wonder why they were in a special support class or why they were different from other children, which caused them to start thinking about themselves.

I could not act as I liked, I could not act freely in a regular class, I did not have any kind of cooperation, so I’m going to feel, oh, I’m different from others.

[Researcher: Did someone say so to you?]

Not that what I really mean… I started thinking about myself and felt I was different on my own after all. (J/15, f)

In addition, some participants experienced new interest in how others saw them:

When I was 5th grade elementary school student, I was talking with a very good teacher, then, from the teacher’s point of view, I felt like understood how others recognized me for the first time. Yes,,, then I was interested in myself. (G/13, f)

In many cases, when participants experienced environmental changes, they became to think about themselves:

When I became a high school student, my relationships also changed anew, and I worried about getting involved with people in a new environment. How do I get used to the environment? I had to look at myself as necessary. (P/18, m)

***Noticing and realizing my own emotions.***

Most participants realized their feelings through relationships with others. Various emotions such as joy, love, gratitude, surprise, frustration, nervousness, contrition, were expressed richly.

(At junior high school) The teacher was very scared, and he got angry immediately. He was a scary person, so I had to check my belongings many times. I would be scolded by the teacher when I forgot something. (I/18, f)

On the other hand, more than half of the participants not to express their emotions but to work things out on their own.

Because they (parents and teacher) only say, “you should try your best first,” or “let’s go as it is for a while.” So I didn’t talk to my mom about something even I had trouble with in school. Well, I thought it was worthless to talk to people, both the counselor and my family. (G/13, f)

***Being able to be myself feel relaxed.***

Most participants felt a sense of security through stable relationships with their friends, classmates, teachers, and family members.

In junior high school, and in high school, well, there was the person who kept company at my talk not to stop, till the end, nevertheless once I started talking, I never finished till all the story ends. I felt relieved at that time. You know, I felt, oh, I am O.K. in this way. (N/ 18, m)

In addition, some participants experienced a stable self through life stability, the improvement of relationships, and making use of their own senses, through the enjoyment of hobbies.

(In elementary school) when I stopped to get angry and violence like beating, kicking…then, the friends gradually increased. Maybe… maybe…naturally. But the results came from junior high school students. Yeah, I really relieved at that time. (E/24, m)

I have loved crafts with cardboard and made models, like a plastic model [laughs]. It still continues now. (B/18, m).

***I can’t accept myself.***

Some participants felt that it was difficult to accept themselves; they thought of themselves as bad people and hated themselves.

Well…I have a point of view that I hate myself who cannot do well like others. Because… people kept pointing out what I could not do well in elementary school and so on. (B/18, m)

***Everyday life is hard; fear and anxiety never go away.***

Most participants had experienced other people’s negative perceptions of their disorders:

I guess that was a strange prejudice, in junior high school. Some classmates thought that someone who needed go to the psychiatry was… a slightly …strange, so I could not tell them that I went to the psychiatry…, I was scared to say. Yes, I’m scared to say now, I have not said yet. (I/18, f)

In addition, many participants felt pressure to do things that they could not do well, and they constantly struggled with academic achievement and relationships with classmates and teachers, which caused daily anxiety and tension.

I think I’m working my brain harder than anyone else. Sometimes others tell me that you should have your brain to rest, but if I will take a little rest and feel relieved, soon it will lead to mistakes, yeah, I know, so until I go to bed, I cannot but do my best. (O/16, m)

Participants also talked about feeling insecure at schools and even at home. Some participants were reluctant to relate to people because of fear and anxiety.

I thought I would make a mistake, and didn’t really say a word anymore. (D/24, f)

Most participants described depleted physical strength in everyday activities like relationships with people, and going to school; some described difficulties with sensory sensitivity:

As I thought, [the sun shine] is too bright. When the weather is fine, it's painful for me, it's like can’t stand it anymore… (O/16, m)

***Realizing my own sensibilities and, my characteristics***

Many participants had realizations about their own characteristics, such as unique ways of thinking or acting in relationships with others.

I didn’t think that it was such a great thing (making a three-dimensional work without drawing), but recently, I was very surprised by my parents. They said they never can do that. I have a picture in my head. (C/16, m)

I was told by my friend that the act I was doing was different from normal, and I clearly understood it was true. I had told so from the friend a few times, from a certain person, not for the first time. For the first time in a junior high school, but,,,yeah, yeah, then I realized it was really true. (M/17, m)

Many participants described other people’s reactions prompting self-realizations that caused them to be proud and gain confidence.

After all, when people ask me to do this work, then, I reply them, yes, sure, I did the work, and someone said to me, “Thank you,”…then I can feel I did the work well. Oh, I did! … I can do… yeah, I did it! (D/24, f)

**Intentionality and self-transformation**

***Realizing transformation of subjective and objective self***

Some participants felt that they had changed in terms of, ways of thinking, behavior, and personality, and other felt their perception of their own characteristics and disabilities had changed.

Wow, I have a strong desire to become ordinary, so I was trying to go to university, but now I think it is not necessary to be ordinary, is it ? Because I was not ordinary there are a world that seems to be visible. There’s not much difference whatever I choose. (B/18, m)

When I decided myself something like this, I guess it was only 4th or 5th grade elementary school. It’s only when my parents divorced, so at that time I was not an adult but I felt like that,…I was leaving my childhood, I couldn’t feel so fluffy well…When I was 6th grade, our family moved to another city, so I moved to a regular class, from the special support level. I feel like I need to get it together… (K/17, m)

Participants considered overcoming past painful experiences as connected to their present selves. Almost half of the participants said they felt blessed with their environment and grateful to their families and the people around them.

Because I’ve been working hard until now, I have confidence, I guess I’m getting stuck, I have experiences, so I can do something with it this way, already my experiences has been utilized well in my life. (P/18, m)

***Creating myself to be the way I want.***

Participants imagined what they wanted to be, such as being on their own and independent from their parents. They wanted to live better and have fun. Their daily life presented various challenges, such as improving academic performance, creating art, and working and earning money.

Well, now, yeah,,, I don’t think that everyone should admit me. Let’s not hide the sickness, well, let’s live in a place where others can accept me as I am. (Q/18, m)

What do I think of life, … life, and after all, I don’t want to make that boring life, I don’t want to make it into boring high school life, I want to make something better. It’s in a better direction, and even if I am not aiming for the best, I say, aiming for the better. (N/18, m)

I had some pets, they were mine, so I had to take care of them. My parents told me it was about time for you to work. Well, I hate to be told so, … and I did my best [to find a job]. (D/24, f)

***Wish to be accepted by others.***

Participants were keen to be accepted by others. They wanted to be normal, and to be considered normal by others:

I also wanted to think that I was an ordinary girl, I also wanted to be thought that by others too. Even though I knew I was different from others, but I do not want to go [psychiatry] because I usually think that it is good to be normal. So, why do I have to go [psychiatry]? [The doctor told me that] I got better, I was glad, but if I got better, do I need to come [to see the doctor], don’t I? (I/18, f)

They sought to be with people, to be involved, to convey their feelings, and to be understood by others:

It is not disgusting…I would like to talk. Even if I can’t take steps with people, there are so many things I want to talk about, such things too. It’s going to be a machinegun talk, but I want to talk. (N/18, m)

Now I have roughly grasped myself, well, when I overdo it, I say, “I am something like this. I am this kind of human being, that,..so could you please to be generous? (M/16, m)

Participants strongly felt the need to consider the feelings of others involved, and they made efforts to observe the reaction of others carefully and to respond appropriately:

I felt like it was in line with everyone’s actions, and because everyone was thinking this, I decided to do something about reading the air, I decided to do something about it… various topics, hobbies, etc. I'm starting to do everything, whether it's all spread, even I hate or I’m not interested in. (C/16, m)

**Unrealized/unnoticed self.**

***It is hard to realize one’s self.***

Some participants felt that their sense of self was uncertain and unstable:

I… I wonder, somewhere, maybe, there is still a gap, likely to collapse, like a domino, like a jenga…something like this, still, shaking well, I think there are still aftershocks, I think maybe. (O/16, m)

In addition, some participants didn’t have the experiences that their emotions arose or shaken even when they interacted with others:

Feeling… I wonder what I felt,,, when I realized that I was different from the children around me… Who cares?? O.K. If I was different, just try to fit others. (J/16, f)

***The uninterested self.***

Some participants did not focus on their own senses and intentions. Some of them talked about not thinking deeply about themselves, acting passively without asking themselves about their own thoughts and intentions:

This may be a characteristic [of ASD], I couldn’t think about it myself before…It’s like I was doing what I was told to do. I think…you usually say, “I want to do the way I like.” But when I was told something to do and then I did this, I felt that it was my duty to do it. I wasn’t disagreeable, I thought, I was going to do it without question, and say, Yes, I am going to do. (A/19, m)

## Discussion

All participants are interested in themselves and realized own emotions and selves through relationships with classmates, teachers, and families. Most participants spoke strongly about not being understood by others, having anxiety and fear of interaction with others, and distrusting others and society. In some cases not expressing their emotions became a natural pattern of behaviors. Ghosh et al. (2016) reports that many parents knew about hyperactivity in children, but were unaware of unstable emotions. They might not pay much attention to their own emotions, or could not easily recall their emotions. In previous studies people with ASD tend to have difficulty to be aware of own emotions (Suda 2018; Kondo and Ozaki 2017). With ADHD, tend to express themselves by symptoms not by their realization (Krueger and Kendall 2001). Another possibility is that they might not actively seek to share their feelings with others. Many participants had strong senses of distrust and disappointment and tension and anxiety trough relationships. It was considered they hesitate to have interaction and share their emotions with others to defend themselves. Nevertheless, many participants were aware of their own senses, characteristics, and recognized the viewpoints and diverse emotions of others through interaction. Having a sense of sharing emotions through interaction is particularly important for adolescents with developmental disabilities (Beppu 2014; Toyota and Iida 2013), it is important for supporting their self-growth. Geanellos (2002) states that growth of self also changes the relationship with others. She shows the importance of encouraging self-transformation by nurturing the “functional self,” the ability to live in harmony with oneself and others. Anxiety, distrust, fear, isolation and despair hinder a sense of trust, respect, comfort, and connection, but reputation, non-authoritative relationships, acceptance, hope, peace of mind, and understanding can develop them. Urasaki et al. (2014) stated the developmental support based on the formation of a reliable trust relationship is the basis of human development and is required even in adolescence. In addition, Takigawa (2013) pointed out that one of the characteristics of people with developmental disabilities is high degree of anxiety and tension and a low dependence on others. He emphasized on that it is important to develop a sense of security through dependence on others. Safe interaction with others is an important part of realizing well-being and stability and is essential for self-growth (Morioka 2015).

Participants gained self-confidence through interaction with others, and through those interactions gained acceptance of their own perceptions, unique characteristics, and sensitivities. Their intentions were consistent with maintaining relationships, such as expressing involvement and thinking and acting so that others would accept them. In other words, while they had difficulty sustaining harmonious relationships through skills such as “reading the air” (picking up on social cues) and collective action, they tried to consider others and transformed themselves. Kawai (2010) describes a core feature of developmental disorders as the difficulty of holding the subjective self; if there is no boundary between oneself and others, people with developmental disorders will adjust their judgment and actions to others forever, but if they are too centered, they can be intrusive. Participants aim to be understood and they start changing their lives and relationships through trial and error. This is an orientation toward the “self with others” described by Stern (1989) and can be regarded as a state in which “the sense of a core of self” and “the sense of a subjective self” are activated through interaction with others.

Sensory sensitivity can be an obstacle to such development, however. Participants describe various perceptions rushing and overflowing, causing them to feel abstracted. Similar experiences are reported by adults with ASD or ADHD (Takahashi et al. 2010; Kobayashi 2017; Niki 2003). People who always overwhelmed by their characteristic perceptions tend to have difficulty realizing their subjective selves. But interestingly more than half of the participants who expressed the concept of “Unrealized/unnoticed self” also expressed “Interest in self and self-realization”. When they touched the thoughts and emotions of others or when they shared their experiences, they realized their selves through interactions. Ayaya (2011) an adult with ASD said “When I share my experiences with others, I feel that I am definitely here”. And she said that an experience in which others approve and give meaning to her senses and behaviors, that is, shared experiences raises a sense of self. Shiomoto (2011) said “The experience of verbalizing one’s thoughts, communicating it to others, and conveying it to others, realization of oneself becomes more certain.” Sharing adolescents’ experiences and senses through interactions based on security and trust supports the development of self. Thus, nursing care focusing on internal experiences and on emotional sharing, stimulates the activation of Stern’s “sense of a core of self”, “sense of a subjective self” and “sense of a verbal self”, and it will support the adolescents’ self-growth in the long term.

## Implications for Nursing

Adolescence is a period of development during which emotional support is especially needed. Care must be taken to ensure that communication does not pose a threat to those who feel anxiety and fear on a daily basis. A study about adolescents’ preferences for caregiver interactions showed that the most important factor in care for them is to realize they that are respected (Schaeuble et al. 2010). The care that allows adolescents to feel respected and safe will decrease their fear and anxiety and support their healthy self-development. It is not possible for adolescents to overcome developmental tasks alone (Sadamoto 2009). Further, a sense of security and self-affirmation cannot be achieved by oneself alone without others (Takayama 2010; Okura 2018). Therefore, efforts should be made to know and respond to the adolescents’ inner world and self. Through daily care, nurse can realize and share their various emotions together. For example, by asking “How do you feel?” to know and share their emotions, it becomes easier for them to pay attention to their own emotions, realize them, and develop the sense of self. It is not enough only through language that we know and share emotions. Because many people with ASD have difficulty in linguistic expression, and some adolescents tend not to talk their emotions to others as this research shows. It is important that nurses always explore adolescents’ emotions based on their facial expressions and mood, unique expression like “irrelevant and metaphorical language” (Kanner 1946), voice intonation and posture, gaze, gestures and physical condition. Further, sharing the child's behavior that the family can identify with nurses is also important.

By sharing the children’s behavior known to the family with the nurse, it may increase nurses’ understanding of children in daily care. In addition, this may help the parent’s understanding of the child emotion and lead for parents to feeling of love for the child, joy and confidence as a parent, we considered that parent–child interaction may be promoted. Nurses are required to have a long-term attitude to explore the meanings and reasons of children’s statements and actions with their families.

An important point in supporting adolescents with developmental disabilities is that they find value in their relationships with others and exist in the world with their independence on the basis of their subjective self (Sasaki 2013; Kawabata 2005). Advancing research and considering support for adolescents with developmental disabilities may lead to a re-evaluation of the values and nature of nursing, that captures and approaches humans as a whole, not by disability or symptoms and that focus on human–environment interaction.

## Limitations and Strengths

This was a self-report survey and interview, there is a possibility of memory bias. Results obtained from a small number of subjects cannot be generalized. However, there are few nursing studies that ask adolescents with developmental disabilities about self by one-to-one interview. This study provided important insights into self-development in adolescents with developmental disabilities by describing in detail their experiences about self. In addition, the fact that adolescents who are not good at communication and are thought to have high interaction anxiety could talk to the researcher about themselves is worth examining. Telling others about self can be an opportunity to grasp self objectively and to think about self and increase self-awareness. Further research clarifying the process of self-development by expanding the target such as people whose linguistic expressions are more limited may contribute to provide new knowledge about self-development. In addition, collaborative research with adolescents may provide useful information for actual, everyday support.
